# The current role of precision surgery in oligometastatic prostate cancer

**DOI:** 10.1016/j.esmoop.2022.100597

**Published:** 2022-10-06

**Authors:** M. von Deimling, P. Rajwa, D. Tilki, A. Heidenreich, M. Pallauf, A. Bianchi, T. Yanagisawa, T. Kawada, P.I. Karakiewicz, P. Gontero, B. Pradere, G. Ploussard, M. Rink, S.F. Shariat

**Affiliations:** 1 Department of Urology, Comprehensive Cancer Center, Medical University of Vienna, Vienna, Austria; 2 Department of Urology, University Medical Center Hamburg-Eppendorf, Hamburg, Germany; 3 Department of Urology, Medical University of Silesia, Zabrze, Poland; 4 Martini-Klinik Prostate Cancer Center, University Hospital Hamburg-Eppendorf, Hamburg, Germany; 5 Department of Urology, University Hospital Cologne, Cologne, Germany; 6 Department of Urology, University Hospital Salzburg, Paracelsus Medical University Salzburg, Salzburg, Austria; 7 Department of Urology, University of Verona, Azienda Ospedaliera Universitaria Integrata, Verona, Italy; 8 Department of Urology, The Jikei University School of Medicine, Tokyo, Japan; 9 Department of Urology, Okayama University Graduate School of Medicine, Dentistry and Pharmaceutical Sciences, Okayama, Japan; 10 Cancer Prognostics and Health Outcomes Unit, Division of Urology, University of Montreal Health Center, Montreal, Canada; 11 Division of Urology, Department of Surgical Sciences, San Giovanni Battista Hospital, University of Studies of Torino, Turin, Italy; 12 Department of Urology, La Croix Du Sud Hospital, Quint-Fonsegrives, France; 13 Hourani Center for Applied Scientific Research, Al-Ahliyya Amman University, Amman, Jordan; 14 Karl Landsteiner Institute of Urology and Andrology, Vienna, Austria; 15 Department of Urology, Weill Cornell Medical College, New York, USA; 16 Department of Urology, University of Texas Southwestern, Dallas, USA; 17 Department of Urology, Second Faculty of Medicine, Charles University, Prague, Czech Republic; 18 Institute for Urology and Reproductive Health, Sechenov University, Moscow, Russia

**Keywords:** prostate neoplasms, oligometastatic, oligometastasis, cytoreductive radical prostatectomy, metastasis-directed therapy, PSMA-PET

## Abstract

Oligometastatic prostate cancer (omPCa) is a novel intermediate disease state characterized by a limited volume of metastatic cells and specific locations. Accurate staging is paramount to unmask oligometastatic disease, as provided by prostate-specific membrane antigen-positron emission tomography. Driven by the results of prospective trials employing conventional and/or modern staging modalities, the treatment landscape of omPCa has rapidly evolved over the last years. Several treatment-related questions comprising the concept of precision strikes are under development. For example, beyond systemic therapy, cohort studies have found that cytoreductive radical prostatectomy (CRP) can confer a survival benefit in select patients with omPCa. More importantly, CRP has been consistently shown to improve long-term local symptoms when the tumor progresses across disease states due to resistance to systemic therapies. Metastasis-directed treatments have also emerged as a promising treatment option due to the visibility of oligometastatic disease and new technologies as well as treatment strategies to target the novel PCa colonies. Whether metastases are present at primary cancer diagnosis or detected upon biochemical recurrence after treatment with curative intent, targeted yet decisive elimination of disseminated tumor cell hotspots is thought to improve survival outcomes. One such strategy is salvage lymph node dissection in oligorecurrent PCa which can alter the natural history of progressive PCa. In this review, we will highlight how refinements in modern staging modalities change the classification and treatment of (oligo-)metastatic PCa. Further, we will also discuss the current role and future directions of precision surgery in omPCa.

## Highlights

• The prevalence of omPCa will increase due to implementation of more accurate imaging modalities such as PSMA-PET.
• The standard of care for omPCa is evolving; PSMA-PET may further accelerate changes in the treatment landscape.
• CRP may confer a survival benefit while improving long-term local symptomology in *de novo* omPCa.
• Ongoing prospective trials will help clarify the role of CRP in the management of *de novo* omPCa.
• RGS may become increasingly important to allow targeted surgical precision strikes such as during tailored SLND.

## Introduction

In developed countries, the prevalence of metastatic prostate cancer (mPCa) constitutes ∼10%-20% of all prostate cancer (PCa) patients, with a recent trend toward an increasing incidence.^1^, ^2^, ^3^, ^4^ mPCa comprises a spectrum of metastatic volume and locations, reflecting the multistep polyclonal nature of cancer spread, colonization, growth, and continuous interplay.^5^ Within this, oligometastatic PCa (omPCa) has gained increasing attention as an early step in this polyconditional natural history; it is in itself characterized by a limited number of metastatic lesions in specific locations.^5^ In these patients, the pace of metastatic progression is one of the most important clinical determinants of hovering time in the oligometastatic state.^6^ Therefore, one may hypothesize that some patients with omPCa, similarly to nonmetastatic PCa, could still be cured if all tumor sites were treated efficiently and effectively. In that regard, treatment decisions are guided by several prognostic and practical parameters such as time, location, and volume of metastatic disease presentation.^7^

In 2020, the European Society for Radiotherapy and Oncology and the European Organization for Research and Treatment of Cancer published a consensus recommendation regarding the classification of oligometastatic disease in general.^8^ An expert panel suggested classifying *de novo* oligometastatic disease as a condition in which patients do not have a history of poly- or oligometastatic disease before the diagnosis of cancer.^8^ Next, depending on the time interval between the primary cancer diagnosis and detection of oligometastatic disease, *de novo* oligometastatic disease is further subdivided into synchronous (<6 months) and metachronous (>6 months after the primary cancer diagnosis) disease states.^8^ Metachronous disease may be referred to as oligoprogressive if patients are under active systemic therapy at the time of oligometastatic disease diagnosis. In contrast, metachronous oligorecurrence refers to patients without systemic therapy at the time of oligometastatic disease diagnosis.^8^

In mPCa, most of the available studies did not incorporate this precise definition but used *de novo* and synchronous mPCa interchangeably to describe a condition in which metastases were detected at the time of PCa diagnosis. Conversely, when the metastatic spread occurred after a local disease state at initial diagnosis, generally after initial treatment with curative intent of the primary tumor, it was interchangeably referred to as recurrent or metachronous mPCa. Furthermore, there is currently no uniform definition of mPCa regarding the metastatic volume and location. It is generally accepted that omPCa refers to a limited number of metastases in bones and/or lymph nodes (LNs) but not in visceral organs, as this site of metastasis is associated with disproportionally worse prognosis.^9^^,^^10^ Most studies employed the Chemohormonal Therapy Versus Androgen Ablation Randomized Trial for Extensive Disease in Prostate Cancer (CHAARTED)’s definition of omPCa, which defined high-volume metastatic disease as the presence of visceral metastases and/or at least four bone lesions with at least one lesion outside of the vertebral column and/or pelvis^11^; all other disease states are considered as low-volume metastatic burden. Of note, this classification merely consists of clinical variables and does not consider the biological underpinnings of the metastases. Moreover, it was based on conventional imaging such as bone scan (BS) and computed tomography (CT), which are now outdated.

With more accurate imaging modalities such as the prostate-specific membrane antigen (PSMA)-positron emission tomography (PET), the omPCa population is likely to become more commonly identified, with a significant number of PCa patients previously thought to have high-risk nonmetastatic PCa being unmasked as having omPCa.^12^ With this shift, the optimal management for omPCa remains to be defined, especially in the era of molecular imaging, as the results of the current omPCa management are based on studies that included patients diagnosed with omPCa on conventional imaging. This leads to a patient shift, allowing for innovative concepts for the first time in mPCa. Moreover, this also results in the prognosis of high-risk nonmetastatic PCa improving due to treatment intensification with reclassification of the omPCa state. The high-risk nonmetastatic patients who remain will also experience better prognosis due to removal of the omPCa patients previously being considered as part of them (Will Rogers phenomenon).

Cytoreductive surgery is a standard treatment in different metastatic cancers.^6^^,^^13^^,^^14^ In omPCa, surgical treatment may consist of primary tumor-directed or metastasis-directed surgery (MDS), both together with tailored systemic therapies. It is, however, essential to realize that both are still experimental today. In this review, we will provide an overview of the surgical treatment landscape in patients with omPCa. Furthermore, we will discuss current changing diagnostics and future concepts in the management of omPCa.

## Diagnostics of oligometastatic prostate cancer

The staging modality significantly affects the time point in the natural history at which the metastatic disease is detected. Conventional imaging for staging localized, high-risk PCa includes CT, BS, and magnetic resonance imaging, but the sensitivity of each of them to detect metastatic disease is limited.^15^ The current staging paradigm in PCa has changed with the introduction of PSMA-PET, which has its utility in staging both primary and recurrent PCa.

Staging patients with high-risk PCa before curative-intent surgery, a recent randomized controlled trial (RCT) assessing PSMA-PET imaging (i.e. the proPSMA trial) found that PSMA-PET had a sensitivity of 85% versus 38% for conventional imaging, which translated into a 27% higher accuracy for detecting metastases.^12^ In subgroup analysis, PSMA-PET was superior to CT/BS in detecting both pelvic nodal disease (91% versus 59%) and distant metastases (95% versus 74%).^12^ Interestingly, 30% of patients with high-risk PCa had pelvic nodal or distant metastatic disease (9% nonregional nodal, 10% bone, 1% visceral metastases).^12^ In another study, PSMA-PET results changed the therapeutic strategy in 29% of men scheduled for radical prostatectomy (RPx) for biopsy-proven PCa.^16^ These findings highlight that patients with localized PCa on conventional imaging may harbor occult (oligo-)metastatic disease that can be uncovered using PSMA-PET.^17^, ^18^, ^19^ The European Association of Urology (EAU) guidelines still do not recommend PSMA-PET as first-line imaging modality, as little is known if the PSMA-PET-based change in therapy leads to better survival outcomes or health-related quality of life (QOL).^15^ Nevertheless, the authors of this review believe that more accurate imaging is a necessary and constructive step toward more precise care and, therefore, should be embraced while learning about its opportunities, pitfalls, and challenges.

In metachronous PCa, the PSMA-PET imaging allows for earlier detection of the location and quantity of metastatic spread. This shift to earlier detection in the natural history significantly impacts the diagnosis, conceptual understanding, and consecutive treatment of omPCa. For example, detection of LN metastases on conventional imaging is currently limited by the latter’s low sensitivity (i.e. ∼40%) and poor performance at low and very low prostate-specific antigen (PSA) levels.^20^, ^21^, ^22^ Similarly, for bone metastases, studies have shown that in patients with biochemical recurrence after primary treatment with curative intent and PSA levels <7 ng/ml, the probability of a positive BS was <5%.^15^^,^^21^^,^^23^ Conversely, the PSMA-PET may localize up to 75% of recurrent PCa with a positive predictive value of 0.84.^18^ Overall, on a per-lesion analysis, sensitivity and specificity of the PSMA-PET were reported to be 75% and 99%, respectively, with almost similar values on a per-patient analysis (77% and 97%, respectively).^24^^,^^25^ However, there is also a PSA-dose-dependent performance of PSMA-PET’s diagnostic accuracy: the percentage of positive PSMA-PET scans at PSA levels >2 ng/ml was 95%, whereas at very low PSA levels <0.2 ng/ml, the pooled estimate of a positive PSMA-PET scan was 33%.^24^ Moreover, the type of primary treatment conferred significantly different PSMA-PET positivity rates of the prostate bed [RPx = 22% versus radiotherapy (RT) = 52%] in biochemically recurrent PCa patients.^24^ Despite this residual ‘blind spot’ at very low PSA levels, the diagnostic accuracy of PSMA-PET is significantly superior to that of conventional imaging modalities. It leads to higher detection rates of oligorecurrent hormone-sensitive PCa (HSPC) at low PSA levels, which previously was a rare disease state.^26^ Whether these patients benefit from the current standard of care (SOC) for metastatic HSPC (mHSPC) detected with conventional imaging remains to be investigated, but it certainly opens the window of opportunity toward achieving cure in mHSPC via local targeted therapies.

The more accurate detection of LN and/or distant metastasis on PSMA-PET is of prognostic importance, thereby challenging current disease classifications and treatment concepts.^26^ In the ORIOLE trial, which randomized patients with omPCa to stereotactic ablative radiotherapy (SABR) versus observation and blinded the investigative team to the PSMA-PET/CT staging results, patients with untreated PET-avid lesions had significantly worse progression-free survival (PFS) at 6 months (38% versus 5%).^27^ The main difficulty of the PSMA-PET’s more accurate and earlier detection of metastatic spread arises from delineating the optimal treatment and follow-up in such patients.^15^ For instance, a recent study reported a change in the intended disease management in nearly two-thirds of the patients with biochemically recurrent PCa, based on PET/CT results.^17^ This supports the logical thought that more accurate diagnostics may help to avoid under- and overtreatment and may allow PSMA-PET-directed metastasis-directed treatment (MDT) due to better classification of the disease state.^28^^,^^29^

Aside from PSMA, several other PET tracers have been evaluated. While recent prospective trials employed choline PET/CT, a growing body of evidence suggests that staging with PSMA-PET/CT is associated with better survival in omPCa management compared to other tracers.^30^, ^31^, ^32^, ^33^, ^34^, ^35^ PSMA is a transmembrane protein that is highly overexpressed on the cell membrane of nearly all prostatic cancer cells and may also correlate with advanced disease stages and castration-resistant prostate cancer (CRPC).^36^ Which radiopharmaceutical ligands perform best in combination with PSMA for which staging purpose is still debated. According to available evidence, the ^68^gallium (Ga)-PSMA- and ^18^fluoride (F)-PSMA-PET/CT performed equally in primary staging and re-staging of biochemical recurrences, whereas ^18^F-PSMA-1007 is superior in identifying local recurrences at PSA levels ranging between 0.5 and 3.5 ng/ml.^37^ Moreover, ^18^F-PSMA-PET may be better for staging the primary prostate tumor or the prostate bed but is at the same time associated with more false-positive bone lesions.^37^ In clinical practice, the choice of the tracer will most likely depend on its availability, the associated cost, the health care system’s reimbursement regulations, and a center’s experience in reading and interpreting the results.

In summary, there are a few critical points to consider in the clinical use of PSMA-PET imaging. PSMA-PET imaging leads to the detection of metastatic disease at earlier stages than ever before, and it results in more accurate classification of M1 stages, which may allow earlier treatment of lesions with a potentially higher likelihood of success. As current guideline recommendations are based on trials that employed conventional imaging, the clinical implications of this stage shift in PCa management will require further evaluation to form our understanding and shape of treatment/monitoring strategies. While PET-directed focal therapy may result in a PSA decline of 50% or more, its effect on long-term survival remains elusive.^18^ On the other hand, a negative PET result increases the chances of actually having a negative test result with some evidence toward significantly better long-term PFS.^28^ Therefore, the optimal timing in managing PSMA-PET-positive lesions warrants prospective evaluation in well-designed trials. In that regard, PSMA-PET imaging may play an important role in guiding MDT and assessing its efficacy as a response marker in addition to liquid biomarkers such as PSA and circulating tumor DNA.^30^^,^^31^^,^^38^ Further refinement of PSMA-PET information is needed to justify the surgical treatment of PSMA-positive regions only.^39^^,^^40^ Also, some uncertainty remains in patients with very low PSA levels <0.5 ng/ml. Additionally, PSMA-PET is insufficient to detect micrometastasis (<5 mm), and the impact of systemic therapy on PSMA-PET imaging results is currently unclear; it is, however, likely to impair its accuracy.^41^

## Treatment of the primary

From a biological standpoint, there is a strong rationale for treating the primary tumor in mPCa. For metastases to leave the primary site, evade detection, travel, pass natural boundaries, settle, survive, and proliferate in a distant organ apart from the primary tumor, the formation of a receptive microenvironment, among others, is necessary.^42^ Tumor-secreted factors orchestrate this process and contribute to a hospitable environment.^43^^,^^44^ Moreover, tumor self-seeding can lead to colonization of circulating tumor cells (CTCs) in their primary tumor of origin.^45^ These polyclonal tumor cells persist despite hormonal/systemic therapy, facilitating disease progression.^46^ Therefore, elimination of the primary tumor can reduce the number of CTCs, the levels of tumor-secreted factors such as cytokines, and the levels of microRNA.^47^ As a result, treatment of the primary tumor may prevent the development of novel metastasis and/or delay the progression of existing metastatic lesions, thereby improving survival outcomes. For example, *in vivo* data from animal models with mPCa showed oncological benefits and decelerated progression when the primary tumor was removed.^48^^,^^49^ Moreover, cytoreductive treatment of the primary tumor has been suggested to enhance and sustain the response to androgen deprivation therapy (ADT),^50^^,^^51^ delaying time to CRPC and reducing ADT-associated side-effects.

### Current evidence for radiotherapy to the prostate as local therapy

For *de novo* low-volume mPCa, RT to the prostate is now considered the SOC, based on the results of two RCTs.^15^ Firstly, the HORRAD trial enrolled men with previously untreated mHSPC with any number of bone metastases and did not find a significant improvement in overall survival (OS) for the intervention group [ADT plus external beam radiation therapy (EBRT); *n* = 216] compared to ADT alone (*n* = 216).^52^ EBRT consisted of 70 Gy in 35 fractions of 2 Gy during an overall treatment time of 7 weeks or 57.76 Gy in 19 fractions of 3.04 Gy three times a week for 6 weeks. Next, in arm H of the STAMPEDE trial, a multi-stage, multi-arm trial, RT to the prostate resulted in a significantly longer OS after 3 years (81% versus 73%; *P* = 0.007) in patients treated for mHSPC with low metastatic burden (according to the definition used in the CHAARTED) but not in the high-volume metastatic subgroup.^53^ RT consisted of 36 Gy in 6 consecutive weekly fractions of 6 Gy or 55 Gy in 20 daily fractions of 2.75 Gy over 4 weeks. Notably, these doses are smaller compared to standard treatment regimens (74-80 Gy) in localized PCa.^15^ A meta-analysis of the two trials did not find a difference in OS and PFS in unselected patients; however, when stratified by the number of bone metastases, patients with four or fewer bone lesions had an absolute improvement in survival of 7% at 3-year follow-up.^54^ A third trial (PEACE-1) will provide further data regarding the benefit of RT to the prostate in low-volume mHSPC, but the number of events in the radiographic PFS and OS subgroup has not yet been reached for final analysis.^55^

### Oncological outcomes of cytoreductive radical prostatectomy in de novo metastatic HSPC

As RT and RPx are equally effective treatment options in localized PCa, it may be assumed that RPx is also a reasonable therapeutic option in omPCa. Evidence supporting cytoreductive radical prostatectomy (CRP) in newly diagnosed mHSPC is currently limited to retrospective, small prospective, or population-based studies. These studies overwhelmingly showed improved survival outcomes [OS, cancer-specific survival (CSS), or PFS] in patients who underwent CRP compared to patients who did not receive local therapy regardless of the metastatic volume.^56^, ^57^, ^58^, ^59^, ^60^, ^61^, ^62^, ^63^ However, systematic comparisons in a pure oligometastatic setting are flawed due to the heterogeneity in study designs, various definitions of oligometastasis, and oncological endpoints assessed. Some studies included M1c patients,^57^, ^58^, ^59^^,^^61^^,^^64^ while others did not.^65^, ^66^, ^67^, ^68^, ^69^

Including all M stages, several population-based studies showed a significant survival benefit for patients undergoing CRP for mPCa compared to patients without local therapy.^57^^,^^59^^,^^61^^,^^62^ When assessing the oncological efficacy of CRP in oligometastatic HSPC (omHSPC), the endpoints of interest are CRPC-free survival and OS. In a case-control study, Heidenreich et al*.* showed significantly better CRPC-free survival and clinical PFS rates in 23 patients who underwent CRP with extended pelvic lymph node dissection (PLND) for low-volume omHSPC compared to patients who received ADT alone.^56^ In the local treatment arm, median CRPC-free survival was 40 months compared to 29 months in the ADT arm.^56^ Similarly, a study derived from the prospective Local Treatment of Metastatic Prostate Cancer (LoMP) registry included 40 men who underwent CRP with extended PLND and 40 men who received SOC for *de novo* mHSPC.^70^ The metastatic volume was classified according to the CHAARTED definition, and CRP was offered if patients were fit to undergo CRP and PCa was considered resectable (<T4).^70^ Median CRPC-free survival was 53 months in the CRP arm compared to 21 months in the control arm. However, CRP was not associated with improved CRPC-free survival on multivariable Cox regression analyses.^70^ Despite its prospective setting, this study was limited by a selection bias and misbalance in metastatic burden; 43% of the entire cohort had high-volume disease with a significant misbalance toward the SOC group (20% versus 65%).^70^

Another case series, including 113 mHSPC patients, of which 88 had low-volume and 25 had high-volume metastatic disease (CHAARTED definition), found a mean CRPC-free survival after CRP of 72 months. Moreover, 5-year OS and CSS rates were 80% and 81%, respectively.^68^ Patients were eligible for CRP if the PCa was fully resectable and if there were no gross retroperitoneal LN metastases, bulky pelvic LN metastases >3 cm, or visceral metastases. Overall, 89% received extended PLND.^68^

Steuber et al*.* carried out a prospective case-control study in omHSPC [fully resectable (≤cT3) tumor, ≤3 bone metastases, absence of visceral metastases].^65^ All patients (*n* = 83) were treated with continuous ADT or maximal androgen blockade after diagnosis and 43 underwent CRP. After a median follow-up of 32.7 months in the CRP group and 82.2 months in the control group, the authors did not find a significant difference in CRPC-free survival (*P* = 0.92) and OS (*P* = 0.25) between the two treatment arms.^65^

As RT is now the SOC treatment of *de novo* low-volume omHSPC, CRP will also have to be assessed against RT to the prostate. Currently, there is no high-quality evidence assessing whether CRP is an equally effective local therapy option to RT. Some retrospective studies reported superior survival outcomes for men undergoing CRP compared to RT to the prostate.^58^^,^^61^ However, these studies included all M stages; comparing CRP and RT in such heterogeneous patient populations is challenging. Knipper et al*.* compared survival outcomes from the STAMPEDE arm H with those of patients with newly diagnosed mPCa with a low metastatic burden and found that 3-year OS in the CRP group was non-inferior compared to RT (91% versus 81%).^53^^,^^66^ Additionally, data from the LoMP registry showed similar results comparing outcomes of 48 patients who underwent CRP with extended PLND to 26 patients who underwent external beam intensity-modulated RT for newly diagnosed low-volume mHSPC.^71^ All patients received SOC in addition to local therapy. Of note, from 2019 onwards, patients with low-volume mHSPC were offered RT as SOC if CRP was not possible or was refused by the patient. Overall, there was no significant difference in 2-year OS and CSS between patients receiving CRP or RT (93% versus 100% for both OS and CSS).^71^

### Perioperative morbidity and local symptom control

Perioperative morbidity is an important quality measure to assess the feasibility of CRP in *de novo* mPCa. Several retrospective studies and the Testing Radical prostatectomy in men with prostate cancer and oligoMetastases to the bone (TRoMbone) trial showed that open and robot-assisted CRP were technically feasible in omPCa.^60^^,^^67^^,^^68^^,^^72^ In the TRoMbone trial, all surgeries were carried out robot-assisted and completed without conversion.^72^ The 90-day complication rate after CRP in the study by Sooriakumaran et al*.* was 20.8%.^67^ Of 106 men who underwent CRP for distant (bone) metastatic PCa, 64.4% were continent. Similarly, in the study by Heidenreich et al*.*, 23.7% experienced minor complications and the overall continence rate was reported at 91.3%.^56^ Overall, perioperative complication rates for CRP ranged between 12% and 34%.^60^^,^^67^^,^^68^^,^^72^ Comparing CRP to RPx in nonmetastatic PCa in a population-based cohort, CRP was associated with slightly higher overall complication rates (14.9% versus 12.3%), but the inherent limitations of population-based databases were a bias.^73^ In a retrospective study that assessed perioperative outcomes in patients undergoing robot-assisted CRP for omPCa, 5.3% of the men experienced Clavien–Dindo classification (CDC) grade III complications but no grade IV or grade V complications.^60^ Moreover, there were no urinary complications in the CRP group, whereas 26.8% of patients in the ADT arm required interventions secondary to disease progression.^60^

Reducing local tumor burden may palliate locoregional symptoms and complications related to invasive tumor growth. Several studies showed that local complication rates were similar or even lower in the surgical local therapy arms than in the ADT/SOC groups.^65^^,^^74^ For instance, Won et al*.* found that >50% of patients without primary tumor treatment experienced local events after developing mCRPC compared to much lower rates with previous local treatment (RP = 20%, EBRT = 47%).^74^ Similarly, Steuber et al*.* found significantly higher local complication rates in patients who did not receive local therapy (CRP 7% versus SOC 35%).^65^

### Ongoing trials

Due to this growing body of evidence, several RCTs were launched and are currently ongoing to evaluate CRP’s feasibility and oncologic efficacy prospectively. Recently, the TRoMbone trial successfully randomized 51 patients with newly diagnosed omPCa to CRP plus SOC versus SOC (ADT ± docetaxel) alone.^72^ The study showed that it is feasible to randomize patients with *de novo* omHSPC to a surgical intervention providing the groundwork for larger trials.^72^ In addition, there was no difference in the QOL between the two groups confirming the results of retrospective studies that did not find a significant difference in QOL outcomes in men undergoing CRP compared to men with localized PCa at the time of surgery.^72^^,^^75^ Several phase II and III trials are currently ongoing to test CRP against SOC and/or RT (Table 1). The results of these trials are urgently awaited to clarify the role of CRP. However, it may not be possible to answer all pending questions. For instance, similar to retrospective studies, most trials accrue all M1 stages.^76^ Therefore, subgroup analyses may be underpowered to detect a significant survival benefit for CRP in patients with low-volume mHSPC. Currently, only three ongoing trials accrue patients with low-volume (≤5 bone lesions) omHSPC.

**Table 1:** Two or more arm trials assessing the oncologic efficacy of cytoreductive radical prostatectomy in (oligo-)metastatic prostate cancer

Study	Identifier	*N*°	Setting	BST/SOC/SST	BST/SOC/SST before randomization/intervention	Inclusion criteria	Definition of (oligo-)metastasis	Primary endpoints	Secondary endpoints	Status
Phase II Trial on Best Systemic Therapy or Best Systemic Therapy (BST) Plus Definitive Treatment (Radiation or Surgery) of the Primary Tumor in Metastatic (M1) Prostate Cancer	NCT01751438	180	• BST versus • BST + CRP/RT to the prostate	NR	6 months (±14 days)	• Histologically or cytologically proven PCa • Castration sensitive • ECOG PS 0 or 1 • Life expectancy >2 years	M1 disease by AJCC staging by bone scan, CT, and/or MRI	PFS	NR	Active, not recruiting
**SWOG 1802**: Phase III Randomized Trial of Standard Systemic Therapy (SST) Versus Standard Systemic Therapy Plus Definitive Treatment (Surgery or Radiation) of the Primary Tumor in Metastatic Prostate Cancer	NCT03678025	1273	• SST versus • SST + CRP/RT to the prostate (± MDRT)	Abiraterone, bicalutamide, degarelix, flutamide, goserelin acetate, histrelin acetate, leuprolide acetate, nilutamide, orchiectomy, triptorelin	22-28 weeks	• Histologically or cytologically proven PCa (adeno) • Castration sensitive • MDRT for up to four sites if completed before randomization	• M1 disease by bone scan, CT, or MRI • Metastatic disease on PET scan only but not conventional imaging or solitary metastases by conventional imaging must be confirmed histologically or cytologically	OS	• OS in SST + CRP versus SST alone in the subset who specify the surgical intent stratification factor • Rate of symptomatic local progression • PFS • PFS in the subsets of patients ± MDRT to oligometastatic sites	Recruiting
**SIMCAP**: Phase 2.5 Multi-Institution Randomized Prospective Clinical Trial Evaluating the Impact of Cytoreductive Radical Prostatectomy Combined With Best Systemic Therapy on Oncologic and Quality of Life Outcomes in Men With Newly Diagnosed Metastatic Prostate Cancer	NCT03456843	190	• BST versus • BST + CRP	ADT ± docetaxel	≥1 month	• Histologically or cytologically proven PCa (adeno) • Castration sensitive • ECOG PS 0 or 1	• M1(a-c) disease by bone scan, CT, MRI, or histological confirmation • If solitary lesion, metastasis confirmed with biopsy or two independent imaging modalities (CT, PET, bone scan, or MRI)	• FFS (PSA progression, clinical progression, radiographic progression, or death from prostate cancer) • In phase III OS^a^	In phase II: • OS • CSS • Overall complication rate • Time to biochemical progression	Recruiting
**IP2-ATLANTA**: Phase II Trial on Additional Treatments to the Local Tumour for Metastatic Prostate Cancer: Assessment of Novel Treatment Algorithms	NCT03763253	• 918 • 25 (PSMA-PET substudy)	• SST versus • CRP/RT + SST (+ MDT) versus • MIAT (HIFU/cryotherapy) + SST (+ MDT) • PSMA-PET/CT substudy	• ADT ± docetaxel or other systemic/local directed treatment including abiraterone or enzalutamide • RT defined as palliative/cytoreductive in high-volume metastases or to mirror STAMPEDE local RT arm in low-volume metastases	≤3 months	• Histologically proven PCa • Castration sensitive • ECOG PS 0-2	Metastatic disease (any T, any N, M1+) of any grade, stage, or PSA level	• PFS (composite outcome) • PSMA-PET substudy: diagnostic accuracy regarding detection of residual disease	• Several QOL measures • Progression on PSA and imaging • Cost-effectiveness	Recruiting
**LoMP II**: Phase II trial on Local Treatment With Radical Prostatectomy (RP) for Newly-diagnosed Metastatic Prostate Cancer (mPCa)	NCT03655886	86	• SST + CRP versus • SST + RT to the prostate bed and pelvis	NR	NR	• Histologically proven PCa • Castration sensitive • ECOG PS 0-2 (2 if related to local PCa symptoms)	Newly diagnosed M1 disease by CT or bone scan	Feasibility of randomization at 48 months	CRPC-free survival	Recruiting
**FUSCC-OMPCa**: An Open-label, Randomized Prospective Phase II Trial of Androgen Deprivation Therapy or Androgen Deprivation Therapy Plus Definitive Treatment (Radiation or Surgery) of the Primary Tumor in Oligometastatic Prostate Cancer	NCT02742675	200	• ADT versus • ADT + CRP/RT to the prostate	Bicalutamide, goserelin acetate, flutamide, leuprolide acetate, triptorelin	NR	• Histologically proven PCa • Age between 18 and 80 years • Castration sensitive • ECOG PS 0-2 (2 if related to local PCa symptoms)	• M1 disease by AJCC staging by bone scan, CT, and/or MRI • Metastatic lesions limited to the lymph nodes or bones (number of lesions ≤5)	PFS	• OS • Time to PSA progression • QOL	Active, not recruiting
Testing Radical Prostatectomy in Chinese Men With Prostate Cancer and oligoMetastases to the Bone	NCT03988686	120	• SOC versus • SOC + CRP	ADT ± other systemic therapies based on current guidelines	NR	• Histologically proven PCa • Age between 19 and 75 years • Castration sensitive • ECOG PS 0-1	• 1-3 skeletal lesions on bone-specific imaging • No visceral metastases	Time to CRPC	QOL	Unknown
A Randomized, Controlled, Multi-Center Clinical Trial to Evaluate the Efficacy and Safety of Prostatectomy for Castration-Naive Oligometastatic Prostate Cancer	NCT04992026	128	• SOC versus • SOC + CRP	ADT plus abiraterone	<9 months	• Histologically proven PCa • Age ≥40 and ≤75 years • Castration sensitive • ECOG PS 0-1 • PSA <2 ng/ml before enrollment • Testosterone level of <50 ng/dl or <1.7 nmol/l during treatment for patients previously treated with surgical castration or ADT • Treatment with ≥6 cycles of abiraterone	• ≤5 metastatic lesions • No visceral metastasis • Diameter of a single lesion ≤5 cm or surface area ≤250 cm^2^	• Time to PSA progression • Time to radiographic progression	• Radiographic PFS • Time to CRPC • Time to PSA remission (≥50%) and time to PSA remission (≥90%) • Time to new anticancer treatment	Recruiting

ADT, androgen deprivation therapy; AJCC, American Joint Committee on Cancer; BST, best systemic therapy; CRP, cytoreductive radical prostatectomy; CRPC, castration-resistant prostate cancer; CSS, cancer-specific survival; CT, computed tomography; ECOG PS, Eastern Cooperative Oncology Group performance status; FFS, failure-free survival; HIFU, high-intensity focal ultrasound; LoMP, Local Treatment for Metastatic Prostate Cancer; MDRT, metastasis-directed radiotherapy; MDT, metastasis-directed treatment; MIAT, minimally invasive ablative therapy; MRI, magnetic resonance imaging; NR, not reported; OMPCa, oligometastatic prostate cancer; OS, overall survival; PCa, prostate cancer; PET, positron emission tomography; PFS progression-free survival; PSA, prostate-specific antigen; PSMA, prostate-specific membrane antigen; QOL, quality of life; RT, radiotherapy; SIMCAP, Surgery in Metastatic Carcinoma of Prostate; SOC, standard of care; SST, standard systemic therapy; SWOG, Southwest Oncology Group.

a If FFS in the control arm improved by ≥30% at 2 years after randomization, a phase III study will start and OS will be the primary endpoint.

Based on the STAMPEDE trial’s arm H results, the g-RAMPP trial (NCT02454543) closed early.^53^ Therefore, to facilitate accrual and account for changes in SOC, trials such as the SWOG 1802 employ an adaptive regimen for their SOC groups based on guideline recommendations and/or metastatic burden. Results of the aforementioned ongoing trials will provide valuable evidence regarding perioperative and functional outcomes, and trials such as the SIMCAP or the SWOG 1802 will even allow a direct comparison of CRP versus RT.

In summary, CRP in mHSPC is feasible and there is growing evidence that CRP confers oncologic benefits, but high-quality data are scarce. Most of the existing studies were subject to a selection bias (e.g. highly selected patients with no comorbidities, healthier CRP group, less metastatic burden in the CRP group), which may have skewed the survival outcomes toward more beneficial results in the local therapy cohorts.^58^, ^59^, ^60^, ^61^^,^^64^ Therefore, the results of ongoing RCTs are awaited to conclusively answer whether there is a role for CRP in the treatment of the primary tumor in mHSPC, how effective CRP is compared to RT to the prostate, and whether the metastatic burden (low versus high volume) impacts survival outcomes after CRP. At present, CRP is associated with a significant improvement in locoregional symptom control. In addition, CRP provides molecular pathologists with sufficient tissue for genetic testing, which is becoming increasingly crucial in metastatic PCa.^77^ Nonetheless, due to the lack of data, RT is still considered the primary local treatment for newly diagnosed, low-volume mHSPC.^9^^,^^15^

## Metastasis-directed surgery

In PCa, metastatic spread does not merely occur in a primary-to-metastasis-directed pattern but also in a metastasis-to-metastasis pattern.^78^ Therefore, to decelerate or even stop further metastatic spread, MDT [MDS and/or metastasis-directed RT (MDRT)] has emerged as part of a multimodal treatment strategy in mPCa. By targeting all detectable metastases, MDT might delay the onset of ADT, prolong PFS, and possibly lead to a cure in highly selected patients with a limited spread of the disease.^6^ In light of this, ADT-free survival (ADT-FS) has emerged as a novel endpoint to evaluate the efficacy of MDT in mHSPC. However, ADT-based combination therapy is considered the standard treatment for patients with newly diagnosed mHSPC constraining the applicability of ADT-FS to oligorecurrent mPCa.^15^ Moreover, the imaging technique used during follow-up strongly influences the evaluation of ADT-FS, and it is still debated whether prolonged ADT-FS confers an improvement in OS. Therefore, ADT-FS may be a surrogate for improved health-related QOL but not for hard oncology endpoints.

According to the EAU guideline, MDT should only be offered to patients who enroll in an RCT or are part of a well-designed prospective cohort study.^15^ Several phase I/II trials testing MDRT and/or MDS in omHSPC provided promising evidence regarding the safety and oncological benefit of MDRT and MDS.^79^ For oligorecurrent mPCa, trials such as the STOMP, ORIOLE, and SABR-COMET trial primarily tested an MDRT approach.^27^^,^^34^^,^^35^

### Oligorecurrent HSPC

#### Salvage therapy of locoregional lymph node recurrences

The primary goals of salvage lymph node dissection (SLND) are to delay the initiation of systemic therapy and, in some cases, help prevent locoregional disease progression with its complications. Overall, data on SLND are rare and limited to mostly retrospective studies.^80^ Also, comparisons across studies and to RT are hampered by the heterogeneity of the study populations regarding localization and volume of metastases, previous or concurrent therapies, and the diagnostic imaging used. Moreover, most studies did not rely on PSMA-PET imaging to detect metastasis.^40^

Overall, growing evidence supports the feasibility and possibly some oncologic benefit to SLND in nodal-only recurrent PCa. With <10% complications CDC grade ≥III, SLND is feasible and well tolerated.^40^ According to a recent systematic review, 5-year OS after SLND for oligorecurrent mPCa was ∼84%, whereas 5-year biochemical PFS rates ranged from 6% to 31%.^40^ A matched-case study, including patients with nodal oligorecurrent mPCa, compared MDT [SLND or stereotactic body radiation therapy (SBRT)] plus SOC to SOC alone (immediate or delayed ADT). With a median follow-up of 70 months, the authors found that 5-year CSS rates (98.6% versus 95.7%) were significantly better in the MDT group.^81^

However, there are also less favorable results.^82^ In a retrospective, multicenter study with long-term follow-up (median 87 months), 10-year CSS and OS rates were 66% and 64%, respectively, and 86% of the men included had biochemical recurrence.^82^ Interestingly, in the same study, 21% of patients did not have positive LN at SLND on final pathology.^82^ This shows the challenge of general conclusions as the imaging and the intervention are likely variable but essential to the success of this intervention.

Despite inaccuracies during staging and pathological examination, these results may also be associated with technical difficulties that arise during surgery due to the localization of LN metastasis. In that regard, radio-guided surgery (RGS) may improve the positive LN yield, thereby increasing the staging accuracy of SLND.^83^^,^^84^ For RGS, a radioactive tracer (e.g. ^99m^Technetium-PSMA) is administered before surgery. Intraoperatively, a gamma probe is used to detect LN uptake of the radioactive tracer and evaluate the surgical field. In a recent cohort study, PSMA-radio-guided SLND was carried out in 364 men with oligorecurrent PCa. Preoperatively, 87% of patients had one or two PSMA-avid and 45% had uni- or bilateral pelvic lesions.^85^ Overall, the authors removed metastatic lesions in 94% of the patients, of which 59% had one or two metastases.^85^ Interestingly, 15% harbored six or more lesions, whereas in 5.8% no metastatic tissue was found.^85^ The 2-year biochemical recurrence-free survival rate was 32%, with a treatment-free survival rate of 58%.^85^

As there are yet no guideline recommendations regarding SLND in nodal recurrent PCa, it remains unclear which patients may benefit from SLND. Pre-operative factors associated with biochemical complete PSA response post-operatively are no extrapelvic LN on PET/CT and a pre-operative PSA level <4 ng/ml.^40^^,^^86^, ^87^, ^88^ Moreover, Fossati et al*.* developed a risk stratification tool based on pre-operative characteristics to facilitate decision making.^88^ Post-operative PSA response and initiation of ADT within 6 months after SLND were associated with better cancer-specific mortality (CSM).^82^ In the future, the ongoing PEACE V-STORM trial that randomizes patients to SBRT or SLND plus 6 months of ADT versus SBRT or SLND plus 6 months of ADT plus whole pelvic RT will provide more insight on which patients may benefit from SLND and the role of MDT in oligorecurrent HSPC in general^89^ (Table 2). In particular, patients will be stratified by the type of MDT (SLND or SBRT) and the type of PET tracer, which will provide further evidence regarding the role of PSMA-PET imaging in staging omPCa.^89^

**Table 2:** Selected trials assessing the oncological efficacy of metastasis-directed treatment in oligorecurrent/oligometastatic prostate cancer

Study	Identifier	*N*°	Setting	MDT	Treatment of the primary	Inclusion criteria	Definition of (oligo-)metastasis	Primary endpoint	Secondary endpoints	Status
**PEACE V**: Phase II trial on Salvage Treatment of OligoRecurrent Nodal Prostate Cancer Metastases (STORM)	NCT03569241	178	• MDT + 6 months of ADT versus • MDT + 6 months of ADT + WPRT (45 Gy in 25 fractions)	SBRT or SLND	RPx, RT or RPx ± prostate bed adjuvant/salvage RT	• Histologically proven PCa; oligorecurrent • Biochemical relapse after radical LT • Nodal relapse in the pelvis on choline, PSMA, or FACBC PET/CT with a maximum of 3 positive nodal lymph nodes (≤5 nodes) • WHO PS 0-1	• Absence of bone or visceral metastases • Absence of lymph node metastases above the aortic bifurcation	Metastases-free survival	• Clinical/biochemical PFS • Acute/late toxicity • QOL • OS/CSS • CRPC-free survival • Time to hormonal treatment • Quality-adjusted life years • Sensitivity/specificity of PET/CT	Recruiting
**PLATON**: Randomized Phase III Trial of Local Ablative Therapy For Hormone Sensitive Oligometastatic Prostate Cancer	NCT03784755	410	• SST + LT to untreated prostate primary (low volume) versus • SST + LT to untreated prostate primary (low volume) + MDT	SBRT and/or surgery to all sites of disease	NR	• Histologically proven PCa; oligometastatic • Stage IV at presentation or relapse after curative-intent therapy • Absence of prior treatment with ADT in the neoadjuvant or adjuvant ≤12 months before randomization • Absence of *de novo* stage IV disease N1 M0 without prior primary-directed treatment with curative intent • ECOG PS 0-1	• M1 disease with ≤5 metastases • ≤3 metastases in any non-bone organ system	FFS	• Radiographic PFS • Metastases-free survival • OS • Adverse events • QOL • Economic aspects	Recruiting
**SPARKLE**: Randomized phase III trial on Metastasis-directed Therapy for Oligorecurrent Prostate Cancer	NCT05352178	873	• MDT versus • MDT + 1 month of ADT • MDT + 6 months of ADT + enzalutamide	SBRT and/or surgery	NR	• Histologically proven PCa; oligorecurrent • Biochemical recurrence defined (PSA >0.2 ng/ml) after RPx + post-operative RT and PSA of 2 ng/ml above the nadir after high-dose RT • WHO PS 0-2	• ≤5 extracranial metastases in any organ • Diagnosed on PSMA-PET/CT/MRI • Nodal (N1) only when accompanied by M1a-c disease (≤5)	Poly-metastatic-free survival	• Metastatic CRPC-free survival • Clinical/biochemical PFS • CSS • OS • Acute/late toxicity • QOL	Recruiting

ADT, androgen deprivation therapy; CRPC, castration-resistant prostate cancer; CSS, cancer-specific survival; CT, computed tomography; ECOG PS, Eastern Cooperative Oncology Group performance status; FACBC, fluorocyclobutanecarboxylic acid; FFS, failure-free survival; LT, local therapy; MDT, metastasis-directed treatment; NR, not reported; OS, overall survival; PCa, prostate cancer; PET, positron emission tomography; PFS, progression-free survival; PS, performance score; PSA, prostate-specific antigen; PSMA, prostate-specific membrane antigen; QOL, quality of life; RPx, radical prostatectomy; RT, radiotherapy; SBRT, stereotactic body radiation therapy; SLND, salvage lymph node dissection; SST, standard systemic therapy; WPRT, whole pelvis radiotherapy.

#### Surgical resection of distant metastases

There is growing evidence that resection of distant metastases might prolong short-term oncological outcomes such as ADT-FS. However, current evidence is mainly based on radiotherapeutic approaches and some bone metastases are certainly not amenable to surgical resection. A few case reports and a limited number of larger series reported on metastasectomy, but the overall evidence is scarce.^90^, ^91^, ^92^

The STOMP trial enrolled 62 oligorecurrent mPCa patients with a PSA relapse after initial, curative-intent treatment of the primary who were randomized to MDRT/MDS or surveillance. All patients had less than three extracranial bone metastases (13 M1b and 1 M1c in the MDT group) on choline PET/CT. MDT consisted of SLND/metastasectomy (six patients; SLND in five and lung metastasectomy in one) or MDRT (SBRT, 25 patients).^35^ After a median follow-up of 64 months, ADT-FS was 34% in the MDT group (MDS or MDRT) and 8% in the surveillance group, respectively (*P* = 0.06). The 5-year CRPC-free survival was longer in the treatment group (76% versus 53%) but without a statistically significant difference (*P* = 0.27).^35^^,^^93^ Interestingly, there was no local or symptomatic progression in the MDT group, whereas poly-metastatic progression was the most common indication for starting ADT in both groups (61% MDT, 55% surveillance).^35^

Battaglia et al*.* included 17 patients with visceral or skeletal oligometastatic (≤3 lesions) recurrent PCa after primary treatment and carried out surgical excision of visceral or skeletal lesions.^94^ The authors found that after repeated MDS sessions and a median follow-up of 43 months, 35% of the patients remained radiographically disease-free at last follow-up.^94^ Of note, 77% of the patients did not receive adjuvant or salvage ADT and 4-year OS was 66%.^94^ One of the largest retrospective studies on the role of MDT assessed CRPC- and ADT-FS in patients who developed oligorecurrent mPCa (≤5 metastatic lesions on imaging) following RPx (±adjuvant or salvage RT) with a serum testosterone level >50 ng/dl.^95^ Participants were treated with MDRT or MDS (RT, SLND, or metastasectomy) and 25 patients (13%) received ≥3 MDTs. The estimated median ADT-FS was 66 months, while the estimated median CRPC-free survival was not reached (mean 117 months).^95^

In summary, while these studies suggest that MDT can prolong CRPC- and ADT-FS, no definitive conclusions regarding MDS can be drawn due to the low number of participants and lack of phase III trials with ADT as a comparison arm. In that regard, the recently launched phase III SPARKLE trial (Table 2) aims to investigate whether the addition of short-term ADT (1 or 6 months) ± androgen receptor-targeted therapy (enzalutamide) to MDRT and/or MDS significantly prolongs poly-metastatic-free survival and/or CRPC-free survival in patients with oligorecurrent HSPC. Currently, no high-quality evidence supports surgical excision of distant metastases as a standardized treatment in oligorecurrent mPCa; therefore, it must be considered an experimental approach.

### De novo metastatic prostate cancer

The current evidence regarding the efficacy of MDS in *de novo* (oligo-)metastatic HSPC is scarce as well.^96^ Most studies employed heterogeneous definitions of the metastatic volume and assessed MDRT.^96^ Overall, there is evidence that a multimodal approach, including surgery, may lead to a sustained response compared to ADT alone. Again, given the STAMPEDE trial’s results, it remains elusive whether MDS combined with ADT and primary-directed RT may provide a survival benefit.

In patients undergoing RPx for *de novo* mPCa (M1a-c) without prior brachytherapy or EBRT, a population-based analysis found that lymph node dissection (LND) at the time of CRP resulted in significantly longer 5-year CSM (80.2% versus 64.6%) and OS (79% versus 56.2%) rates compared to RPx alone.^97^ Furthermore, perioperative complication rates were equal between the two arms.^97^ When stratified by M stage, CSM was significantly better in the RPx plus LND M1b subgroup (79.1% versus 64.1%; *P* < 0.001), highlighting that LND may confer a survival benefit even in the presence of bone metastasis.^97^

In a prospective pilot study, O’Shaughnessy et al*.* enrolled 20 *de novo* omHSPC patients with ≤10 node or bone metastases. Patients underwent a multimodal approach including ADT, CRP with PLND, and, if appropriate, retroperitoneal LND as well as SABR. Of note, the primary endpoint was defined as undetectable PSA (<0.05 ng/ml) after testosterone recovery. At 20 months after MDT, all four patients who reached the primary endpoint had M1b disease, again supporting the idea that LND may provide a benefit in the presence of non-nodal metastasis.^98^ Seventy-five percent of patients had PSA levels <0.05 ng/ml after ADT and surgery, while another 20% achieved undetectable PSA through the combination of ADT, surgery, and SABR.^98^

Prospective trials will need to evaluate the oncologic benefit of concurrent MDT at the time of primary-directed therapy in *de novo* mHSPC. The ongoing SWOG 1802 trial randomizes patients with omPCa to standard systemic therapy plus primary-directed therapy or standard systemic therapy alone. Participants are allowed to receive MDRT to up to four disease sites before randomization. As a secondary objective, the investigators plan to assess PFS between patients with and without MDRT (Table 1).

In conclusion, regarding soft oncological endpoints such as ADT-FS, there is growing evidence of the oncological benefit of MDS for both oligorecurrent and *de novo* mHSPC. Still, long-term OS/CSS data are missing. One of the major challenges remains to identify optimal candidates for MDS to avoid overtreatment in patients with short life expectancy and without symptoms related to metastases. MDT will likely consist of a multimodal approach, as such strategies seem to carry the most significant oncological benefit. As long as there are no uniform recommendations, treatment decisions should be made in an interdisciplinary setting based on modern imaging such as PSMA-PET and patients’ life expectancy and preferences. Future work should be directed toward identifying the optimal timing of MDT in oligometastatic (recurrent) disease and the best combination of MDS with MDRT and ADT.

## Summary

Due to the improved staging, omPCa will become a more prevalent disease entity, both at primary diagnosis and in the oligorecurrent setting. Retrospective and small prospective cohorts suggest that surgery (e.g. CRP, SLND, metastasectomy) offers favorable short-term oncologic outcomes combined with local disease control. Nevertheless, there is a lack of high-quality data on oncological outcomes after surgical treatment in omPCa. For *de novo* omHSPC, several trials are currently investigating the oncological efficacy of surgical therapy of the primary tumor. In metachronous metastatic disease, SLND should only be applied in well-selected patients in a multimodal approach to eliminate all detectable disease and prolong PFS. Moreover, in omPCa, growing evidence suggests that treating every metastatic site to reach no evidence of disease improves survival. Consensus efforts need to be made to standardize terminology, capitalize on the full potential of PSME-PET imaging, combine imaging with molecular biomarkers to risk stratify each case, refine surgical/radiation local therapy, and integrate novel therapeutics such as radiopharmaceutical-driven theranostics.
